# A Cross‐Sectional Study on the Status of Female Lung Cancer Patients With Bone Metastasis With or Without Osteoporosis

**DOI:** 10.1002/hsr2.71605

**Published:** 2025-11-30

**Authors:** Pan Luo, Xianli Liao, Ya Chen, Ming Fu

**Affiliations:** ^1^ Department of Respiratory Disease, Daping Hospital Army Medical University Chongqing P.R. China

**Keywords:** lung cancer bone metastasis, osteoporosis, pain level, psychological distress, women

## Abstract

**Background and Aims:**

Late‐stage lung cancer (LC) often leads to distant metastasis to the skeletal system. Furthermore, there is a probability of 32.1% of osteoporosis development in women over the age of 50. Both bone metastases (BM) and osteoporosis cause bone pain. However, research is still limited on the differences in pain and psychological distress between LC patients with and without osteoporosis. Therefore, we aimed to investigate the differences in pain and psychological distress levels among female LC patients with and without BM and osteoporosis.

**Methods:**

The study focused on the incidence of BM in female LC patients (aged 36–87 years and untreated) with or without osteoporosis. A prospective research analysis was conducted to examine the specificity of pain and psychological distress. BM was diagnosed according to the guidelines for BM in patients with LC and confirmed through whole‐body bone imaging and PET‐CT. Osteoporosis was diagnosed according to “Guidelines for the Diagnosis and Treatment of Primary Osteoporosis” (2017), which recommend bone density measurement for confirmation.

**Results:**

Patients with LC (*n* = 176, 59.4%), with LC and BM (*n* = 82, 27.7%), with LC without BM but with osteoporosis (*n* = 12, 4.1%), and with LC and BM in combination with osteoporosis (*n* = 26, 8.8%) were included. Importantly, the incidence of osteoporosis in BM group was greater than that without BM. According to the multivariate regression, LC patients with both BM and osteoporosis experienced more severe pain and higher levels of anxiety and depression than those with BM alone.

**Conclusion:**

This study revealed differences in pain levels and psychological distress between female LC patients with BM and those with BM complicated by osteoporosis. These findings underscore the need for integrated clinical management strategies, such as implementing routine osteoporosis screening for this population and developing tailored pain management and psychosocial support protocols to improve overall care outcomes.

## 1 Introduction

Primary bronchial lung cancer (referred to as lung cancer) is a malignancy with the highest incidence and mortality rates worldwide [1, 2, 3]. According to the GLOBOCAN 2020 cancer incidence and mortality estimates published by the International Agency for Research on Cancer (IARC), lung cancer remains a leading cause of cancer‐related deaths, with an estimated 1.8 million deaths in 2020 [4].

In recent years, research related to the bone metastasis of lung cancer and its complications has received increasing attention [5, 6]. According to clinical data, approximately 30%–40% of patients with advanced lung cancer experience bone metastasis during the course of their disease [7], which has a significant negative impact on both the incidence and survival rates. Bone metastasis not only leads to the destruction of skeletal structures but is also often accompanied by pain, functional impairment, pathological fractures, and spinal cord compression, among other skeletal‐related events (SREs) [8, 9]. These factors increase the complexity of treatment and significantly increase the overall medical costs for patients with bone metastasis [10].

Osteoporosis, a systemic skeletal disease characterized by reduced bone mass and microstructural deterioration [11], poses a significant global health burden, particularly among women. Its incidence rises markedly with age, affecting a substantial proportion of postmenopausal women due to estrogen deficiency. Approximately 71% of osteoporotic fractures occur in women aged 50 years and older [12]. This condition itself is a well‐established cause of chronic pain, primarily from microfractures and deformities, and is frequently associated with anxiety and depression due to functional limitations, fear of fractures, and reduced quality of life [12]. The prevalence of osteoporosis is notably higher in cancer patients, including those with lung cancer. This increased risk is attributed to shared risk factors, the catabolic effects of the cancer itself, and adverse effects of anticancer therapies, including chemotherapy and the use of corticosteroids [13]. Furthermore, the presence of bone metastases exacerbates bone loss, creating a detrimental synergy that makes the co‐occurrence of osteoporosis and bone metastasis particularly common and severe in this population [14].

When patients with lung cancer and bone metastasis also have osteoporosis, bone pain symptoms are often exacerbated and prolonged, increasing the occurrence of skeletal‐related events, such as pathological fractures. This not only results in functional disabilities but also severely affects the quality of life of patients, further leading to psychological symptoms such as depression and anxiety [15, 16]. Additionally, lung cancer bone metastasis combined with osteoporosis is not an isolated condition; in their comprehensive assessment, health care professionals should also consider factors such as the economic burden and social challenges [17, 18]. Therefore, early screening and individualized treatment measures for these patients are especially important and require health care providers to shift from the traditional doctor–patient relationship model to a biopsychosocial medical model, thereby adopting a comprehensive approach that emphasizes both physical and mental health [19].

The literature on pain and psychological distress has focused primarily on cancer patients as a whole [20, 21], with few studies providing detailed analyses of specific populations, such as female lung cancer patients. In consideration of the distinct pathophysiology of osteoporosis in women, particularly in relation to hormonal changes and elevated fracture risk, this study focuses specifically on female patients to facilitate a more comprehensive understanding of the synergistic effects of bone metastasis and osteoporosis in this vulnerable subgroup. Therefore, this study aimed to explore the clinical status of female lung cancer patients with or without bone metastases and osteoporosis. By statistically analyzing information such as numerical pain scores and self‐assessment scales for anxiety and depression, we investigated the differences in pain levels and psychological symptoms between the two groups. This study further aimed to provide a scientific basis for clinical decision‐making, optimize treatment strategies, and improve nursing follow‐up work. The findings are reported below.

## 2 Patients and Methods

### 2.1 Study Population

This study included 296 female lung cancer patients who were newly diagnosed between January 2020 and May 2023 and comprised 254 patients with adenocarcinoma, 20 patients with squamous cell carcinoma, 15 patients with small cell lung cancer, and 7 patients with other types of lung cancer. All the patients were diagnosed and treated at the Department of Respiratory and Critical Care Medicine at the authors' institution. The inclusion criteria for this study were as follows: (1) female patients diagnosed with lung cancer through cytology or histology; (2) bone metastasis confirmed by bone scintigraphy, computed tomography (CT), PET‐CT, or magnetic resonance imaging (MRI); and (3) osteoporosis diagnosed through bone density measurement. The exclusion criteria were as follows: (1) patients with bone diseases other than bone metastasis and osteoporosis; (2) patients with communication barriers that hindered accurate expression of their thoughts; (3) patients with concurrent or metachronous other malignant tumors; (4) patients whose pain, anxiety, and depression were deemed unrelated to lung cancer; and (5) patients who refused further treatment. All patients provided informed consent, and this study was approved by the hospital′s ethics committee. This study has been approved by the Ethics Committee of the Army Specialized Medical Center of the People's Liberation Army of China (Approval Number: 2022236). All research was conducted in accordance with the relevant guidelines and regulations.

## 3 Methods

The baseline for investigation was established based on the initial hospitalization of each newly diagnosed patient. Before the administration of any treatment, data were collected using the Numerical Rating Scale (NRS) as recommended by the Chinese Expert Consensus on the Application of Pain Assessment Scales (2020), the Self‐Rating Anxiety Scale (SAS) [22], and the Self‐Rating Depression Scale (SDS) [23] through questionnaire‐based assessments.

### 3.1 Observed Indicators

(1) To assess the differences in pain between lung cancer patients with bone metastasis and osteoporosis patients, pain was assessed via a numerical rating scale (NRS), with 0 indicating no pain, 1–3 indicating mild pain, 4–6 indicating moderate pain, and 7–10 indicating severe pain, with higher scores indicating more severe pain. (2) The Self‐Rating Anxiety Scale (SAS), which includes 20 items, was used to evaluate the anxiety symptoms in patients. The scores for the 20 items were added and multiplied by 1.25 to obtain the standard score, and the total score was 100. A SAS standard score < 50 was defined as normal, a score 50–59 was defined as mild anxiety, a score 60–69 was defined as moderate anxiety, and a score > 69 was defined as severe anxiety. The higher the score was, the more serious the anxiety symptoms were. (3) The Self‐Rating Depression Scale (SDS), which includes 20 items, was used to evaluate the depressive symptoms in patients. The scores of the 20 items were summed and multiplied by 1.25 to obtain the standard score, and the total score was 100 points. An SDS standard score < 53 was defined as normal, a score 53–62 was defined as mild depression, a score 63–72 was defined as moderate depression, and a score of more than 72 was defined as severe depression. The higher the score was, the more serious the depressive symptoms were.

### 3.2 Statistical Analysis

Two researchers independently entered all data, which were then verified for accuracy before analysis. Statistical analyses were conducted using R software (version 4.2; R Foundation for Statistical Computing, Vienna, Austria).

The primary analyses, including comparisons of pain score changes across predefined patient groups, were pre‐specified. Exploratory analyses were also performed to assess the potential influence of comorbid osteoporosis (defined by bone mineral density measurements) on patient‐reported pain (NRS), anxiety (SAS), and depression (SDS) using multivariate logistic regression. Given their exploratory nature, *p* values from these analyses should be interpreted with caution, and findings are considered hypothesis‐generating for future research.

The association between lung cancer bone metastasis and osteoporosis was evaluated with a multivariate logistic regression model, where osteoporosis status served as the dependent variable. Independent variables included bone metastasis (presence/absence), age (continuous), pathological type (adenocarcinoma, squamous cell carcinoma, small cell carcinoma, or other), and clinical stage (categorical, I–IV). Adjusted odds ratios (aOR) with 95% confidence intervals (CI) for the presence of bone metastasis were estimated using linear contrast methods.

To examine the impact of osteoporosis on pain, anxiety, and depression among patients with bone metastases, separate multivariate logistic regression models were fitted for each outcome: moderate‐to‐severe pain, anxiety, and depression. All models were adjusted for osteoporosis status, age, pathological type, and clinical stage. An interaction term between bone metastasis and osteoporosis was included to enable precise estimation of the osteoporosis effect within the bone metastasis subgroup. Adjusted ORs and 95% CI for comparisons between patients with and without osteoporosis were derived using linear contrasts.

A two‐sided *p* value < 0.05 was considered statistically significant for all analyses.

## 4 Results

### 4.1 Demographic Characteristics of the Study Population

The demographic characteristics of patients with lung cancer with or without bone metastasis and/or osteoporosis are shown in Table 1.

**Table 1:** The demographic characteristics of patients with lung cancer associated with bone metastasis and/or osteoporosis.

	LC BoM−	LC BoM+	LC Osteo+	LC BoM+ Osteo+
Total	176	82	12	26
Age				
＜65	104/176 (59.1%)	50/82 (61.0%)	(58.3%) (7/12)	14/26 (53.8%)
≥ 65	72/176 (40.9%)	32/82 (39.0%)	(41.7%) (5/12)	12/26 (46.2%)
Pathological type				
AC	148/176 (84.1%)	69/82 (84.2%)	(100.0%) (12/12)	25/26 (96.2%)
SCC	13/176 (7.4%)	6/82 (7.3%)	(0.0%) (0/12)	1/26 (3.8%)
SCLC	10/176 (5.7%)	5/82 (6.1%)	(0.0%) (0/12)	0/26 (0.0%)
Other	5/176 (2.8%)	2/82 (2.4%)	(0.0%) (0/12)	0/26 (0.0%)
Stage				
I	38/176 (21.6%)	0/82 (0.0%)	(8.3%) (1/12)	0/26 (0.0%)
II	20/176 (11.3%)	1/82 (1.2%)	(0.0%) (0/12)	0/26 (0.0%)
III	45/176 (25.6%)	4/82 (4.9%)	(33.3%) (4/12)	0/26 (0.0%)
IV	73/176 (41.5%)	77/82 (93.9%)	(58.3%) (7/12)	26/26 (100.0%)
BFAEs	1/176 (0.6%)	(1/82 (1.2%)	(0.0%) (0/12)	1/26 (3.8%)

Abbreviations: AC, adenocarcinoma; BFAEs, bone‐related adverse events; BoM, bone metastasis; LC, lung cancer; osteo, osteoporosis; SCC, squamous cell carcinoma; SCLC, small cell lung cancer.

### 4.2 Comparison of the Incidence of Osteoporosis in Lung Cancer Patients With Bone Metastases

This study included a total of 296 female patients who were newly diagnosed with lung cancer and categorized into four groups on the basis of the presence of bone metastases and osteoporosis as follows: Group A consisted of patients with lung cancer only; Group B included patients with lung cancer and bone metastases without osteoporosis; Group C included patients with lung cancer and osteoporosis without bone metastases; and Group D consisted of patients with lung cancer, bone metastases, and osteoporosis. Among these groups, Group A had 176 patients (59.4%), Group B had 82 patients (27.7%), Group C had 12 patients (4.1%), and Group D had 26 patients (8.8%). According to the multivariate logistic analysis, the results showed that the odds of developing osteoporosis in lung cancer patients with bone metastasis were 3.018 times higher than in those without bone metastasis (95% CI: 1.34–7.352; *p *= 0.01; see Table 2). The proportion of osteoporosis in the bone metastasis group (27.7%) was higher than in the non‐bone metastasis group (8.8%).

**Table 2:** Multivariable logistic regression analysis of the association between bone metastasis and osteoporosis in patients with lung cancer.

		Osteo		Total	OR (95% CI)	*p* value
		Without	With			
BoM	Without	176/296 (59.4%)	12/296 (4.1%)	188/296 (63.5%)	3.018 (1.34–7.352)	0.01
	With	82/296 (27.7%)	26/296 (8.8%)	108/296 (36.5%)		
Total		258/296 (87.1%)	38/296 (12.9%)	296 (100.0%)		

Abbreviations: BoM, bone metastasis; CI, confidence interval; OR, odds ratio; osteo, osteoporosis.

### 4.3 Relationship Between Pain and Osteoporosis in Patients With Bone Metastases From Lung Cancer

**Figure 1:**
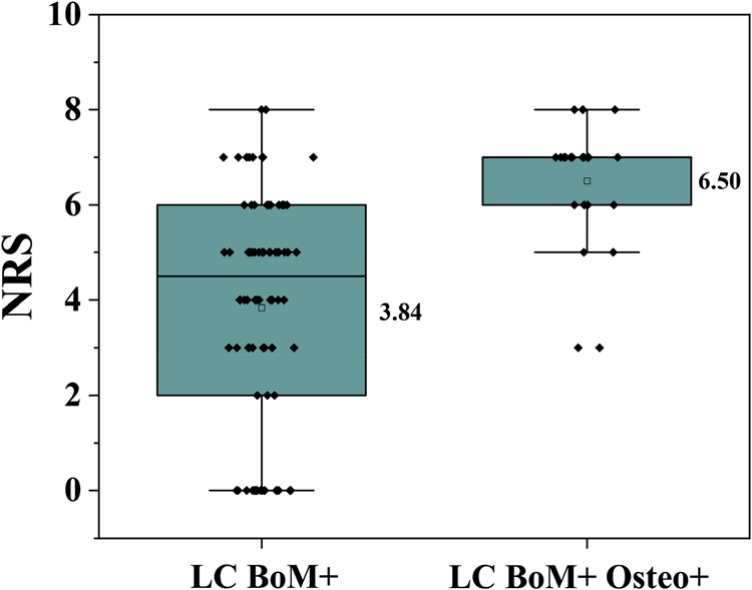
The box plot of NRS scores for Group “LC BoM+” and Group “LC BoM+ Osteo+”.

Based on the patient self‐assessment, the pain levels were categorized into four grades as follows: none, mild, moderate, and severe. In Group B, the number of patients without pain was 18 (22.0%) compared with 0 (0.0%) in Group D. The number of patients with mild pain was 12 (14.6%) in Group B versus 2 (7.7%) in Group D. For moderate pain, the numbers were 42 (51.2%) patients in Group B and 7 (26.9%) patients in Group D. Severe pain was reported by 10 (12.2%) patients in Group B and 17 (65.4%) patients in Group D. The mean NRS score was 3.84 ± 2.42 (95% CI: 3.32–4.36) in patients with lung cancer bone metastasis, while patients with both lung cancer bone metastasis and osteoporosis showed a higher mean NRS score of 6.50 ± 1.30 (95% CI: 6.00–7.00) (see Figure 1). Multivariate logistic regression analysis was performed to ascertain the relationship between osteoporosis and pain in patients with lung cancer and bone metastasis. The analysis revealed that, following adjustment for age, pathological type and clinical stage, the odds of experiencing pain in lung cancer patients with both bone metastasis and osteoporosis were 7.29 times higher than those in lung cancer patients with bone metastasis alone (95% CI: 1.58–33.70, *p *= 0.01; see Table 3). Furthermore, a greater proportion of patients with severe pain were observed in the osteoporosis group compared to the group without osteoporosis (65.4% vs. 12.2%).

**Table 3:** Multivariable logistic regression analysis of the association between osteoporosis and pain in patients with lung cancer and bone metastasis.

		Pain level				Total	OR (95% CI)	*p* value
		None	Mild	Moderate	Severe			
Osteo	Without	18/82 (22.0%)	12/82 (14.6%)	42/82 (51.2%)	10/82 (12.2%)	82 (100%)	7.29 (1.58– 33.70)	0.01
	With	0/26 (0.0%)	2/26 (7.7%)	7/26 (26.9%)	17/26 (65.4%)	26 (100%)		
Total		18/108 (16.7%)	14/108 (12.9%)	49/108 (45.4%)	27/108 (25.0%)	108 (100%)		

Abbreviations: CI, confidence interval; OR, odds ratio; osteo, osteoporosis.

### 4.4 Relationship Between Anxiety Levels in Lung Cancer Patients With Bone Metastasis and Osteoporosis

**Figure 2:**
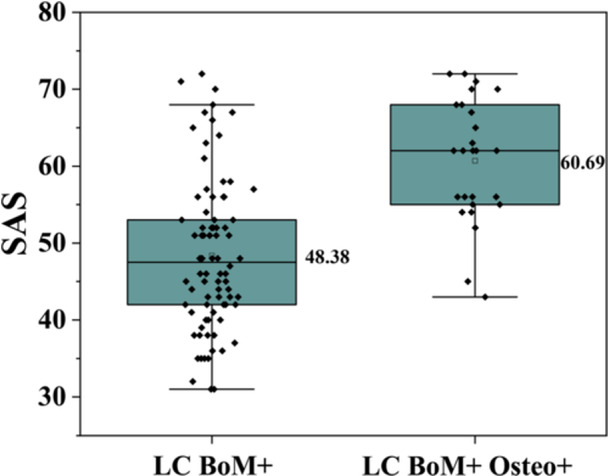
The box plot of SAS scores for Group “LC BoM+” and Group “LC BoM+ Osteo+”.

Based on the patient self‐assessment, anxiety levels were categorized into four grades as follows: none, mild, moderate, and severe. The numbers of patients without anxiety in Group B and Group D were 47 (57.3%) and 2 (7.7%), respectively. The number of patients with mild anxiety was 24 (29.3%) versus 9 (34.6%), whereas the number of patients with moderate anxiety was 8 (9.8%) versus 10 (38.5%). The number of patients with severe anxiety was 3 (3.6%) versus 5 (19.2%). The mean SAS score for lung cancer patients with bone metastasis was 48.38 ± 9.88, with a 95% CI of 46.24–50.52. In contrast, lung cancer patients with bone metastasis complicated by osteoporosis had a mean SAS score of 60.69 ± 7.98, with a 95% CI of 57.62–63.76 (see Figure 2). The findings of the multivariate logistic regression analysis suggest that, after adjustment for age, pathological type, and clinical stage, patients with lung cancer bone metastases complicated by osteoporosis exhibit an 8.96‐fold higher odds ratio of developing anxiety symptoms in comparison to patients with lung cancer bone metastases alone (95% CI: 3.15–25.50, *p *< 0.001; see Table 4), with a greater proportion of severe anxiety than in those without osteoporosis (19.2% vs. 3.6%).

**Table 4:** Multivariable logistic regression analysis of the association between osteoporosis and anxiety levels in patients with lung cancer and bone metastasis.

		Anxiety Level				Total	OR (95% CI)	*p* value
		None	Mild	Moderate	Severe			
Osteo	Without	47/82 (57.3%)	24/82 (29.3%)	8/82 (9.8%)	3/82 (3.6%)	82 (100%)	8.96 (3.15–25.50)	< 0.001
	With	2/26 (7.7%)	9/26 (34.6%)	10/26 (38.5%)	5/26 (19.2%)	26 (100%)		
Total		49/108 (45.4%)	33/108 (30.5%)	18/108 (16.7%)	8/108 (7.4%)	108 (100%)		

Abbreviations: CI, confidence interval; OR, odds ratio; osteo, osteoporosis.

### 4.5 Relationship Between Depression Levels and Osteoporosis in Lung Cancer Patients With Bone Metastases

**Figure 3:**
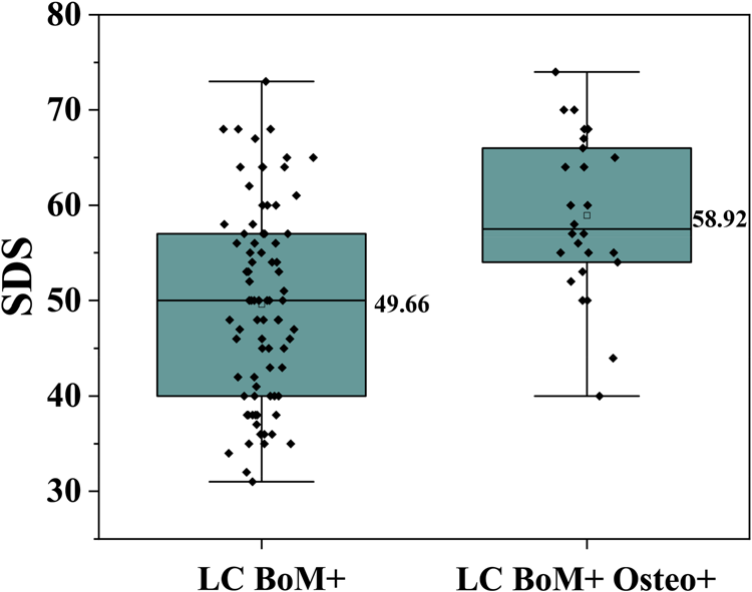
The box plot of SDS scores for Group “LC BoM+” and Group “LC BoM+ Osteo+”.

Based on the patient self‐assessment, depression levels were categorized into four grades as follows: none, mild, moderate, and severe. The numbers of patients without depression in Group B and Group D were 49 (59.8%) and 5 (19.2%), respectively. The number of patients with mild depression was 22 (26.8%) versus 11 (42.3%), that with moderate depression was 10 (12.2%) versus 9 (34.6%), and that with severe depression was 1 (1.2%) versus 1 (3.9%). Lung cancer patients with bone metastasis and comorbid osteoporosis reported significantly higher psychological distress, with a mean SDS score of 58.92 ± 8.38 (95% CI: 55.70 ± 62.14), whereas the mean SDS score was 49.66 ± 10.15 (95% CI: 47.46–51.86) in those with bone metastasis alone (see Figure 3). According to the multivariate logistic regression analysis, patients with bone metastasis and osteoporosis from lung cancer exhibit more severe depressive symptoms(OR = 4.49, 95% CI: 1.55–13.00, *p *= 0.006; see Table 5).

**Table 5:** Multivariable logistic regression analysis of the association between osteoporosis and depression levels in patients with lung cancer and bone metastasis.

		Depression level				Total	OR (95% CI)	*p* value
		None	Mild	Moderate	Severe			
Osteo	Without	49/82 (59.8%)	22/82 (26.8%)	10/82 (12.2%)	1/82 (1.2%)	82 (100%)	4.49 (1.55–13.00)	0.006
	With	5/26 (19.2%)	11/26 (42.3%)	9/26 (34.6%)	1/26 (3.9%)	26 (100%)		
Total		54/108 (50.0%)	33/108 (30.6%)	19/108 (17.6%)	2/108 (1.8%)	108 (100%)		

Abbreviations: CI, confidence interval; OR, odds ratio; osteo, osteoporosis.

## 5 Discussion

With the continuous advancement of research related to lung cancer, an increasing number of health care professionals are focusing on the study, diagnosis, and treatment of comorbidities associated with lung cancer [24]. Owing to the insidious onset of lung cancer, approximately 50% of cases are diagnosed at an advanced stage (stage IV) [25, 26], with bone metastasis being one of the primary sites of hematogenous spread. Among patients with lung cancer bone metastases, 50% present with clinical symptoms, with bone pain being the most significant clinical manifestation [27]. This study focused on female patients, particularly postmenopausal elderly women, who face a greater risk of osteoporosis due to accelerated bone loss [28]. Given that these patients have a baseline condition of lung cancer with bone metastasis, their pain symptoms and psychological distress, whether or not they are accompanied by osteoporosis, should receive increased attention from health care professionals, and active treatment of comorbidities is warranted.

This study first compared the incidence and severity of pain between lung cancer patients with bone metastasis and those with bone metastasis in combination with osteoporosis. Among the 82 female lung cancer patients with bone metastasis, 42 experienced bone pain, resulting in a pain incidence rate of 51%. All 26 lung cancer patients with bone metastasis in combination with osteoporosis reported bone pain symptoms. The study revealed that lung cancer patients with both comorbidities had a greater incidence and severity of pain than did those with bone metastasis only, with a greater proportion of severe pain in the comorbid group. This may be closely related to the pathophysiological changes caused by osteoporosis, such as weakened bone structure and increased susceptibility to fractures, which not only increase the risk of serious complications, such as pathological fractures, but also pain levels in patients [11]. It should be noted, however, that these findings are derived from a single‐center study with a limited sample size; future multi‐center studies with larger cohorts are needed to enhance the generalizability of these results.

Pain can severely impact patients' sleep, mood, and daily living [29]. Pain, discomfort, activity limitations, or complications arising from lung cancer bone metastasis, with or without osteoporosis, significantly affect patients' prognosis and quality of life [30, 31]. Patients often experience fear upon being diagnosed with lung cancer and bone metastasis, whether they are accompanied by osteoporosis or not, which is compounded by intense pain and the risk of bone‐related adverse events, leading to anxiety regarding prognosis and survival. Furthermore, the management of comorbidities can increase the financial burden on patients, further intensifying their psychological distress. Therefore, this study utilized the SAS and SDS to assess patients' anxiety and depression levels and revealed that lung cancer patients with bone metastasis in combination with osteoporosis presented higher anxiety levels than those with bone metastasis only did, particularly highlighting the prevalence of moderate to severe anxiety. Additionally, depressive symptoms in patients with lung cancer bone metastasis and osteoporosis were more pronounced. This finding is consistent with those of related studies, indicating that pain not only causes physiological dysfunction but also triggers stress responses, such as increased catecholamine levels, which in turn exacerbate the perception of pain, creating a vicious cycle of psychosomatic interactions [32]. These findings further underscore the importance of addressing patients' mental health during treatment.

The results of this study indicate that lung cancer patients with bone metastasis in combination with osteoporosis experience greater pain symptoms and higher levels of anxiety and depression than do those with bone metastasis only. In this context, the promotion of a biopsychosocial medical model becomes particularly important, as it encourages health care professionals to consider patients' physical and mental health from a comprehensive and multifaceted perspective to achieve integrated care. To address the complex needs of this population, concrete dual‐management strategies are recommended, including the integration of routine osteoporosis assessment (such as bone density scans and fracture risk evaluation) into standard BoM care protocols, combined use of bone‐modifying agents and personalized analgesia, as well as structured psychosocial interventions such as cognitive‐behavioral therapy or support groups tailored for patients with multiple comorbidities.

Nurses, as the primary caregivers in patients' daily care, play crucial roles in identifying patients' psychological needs, monitoring symptom changes, and facilitating communication between patients and health care providers. Nurses are an indispensable component for improving overall treatment outcomes and quality of life for patients. Therefore, for patients with lung cancer bone metastasis in combination with osteoporosis, comprehensive intervention strategies should be developed that emphasize not only tumor treatment but also the management of osteoporosis and psychological support for optimal patient care outcomes.

## Author Contributions

**Pan Luo:** conceptualization, data curation, formal analysis, methodology, and writing – original draft. **Xianli Liao:** conceptualization, data curation, formal analysis, and investigation. **Ya Chen:** project administration, resources, and supervision. **Ming Fu:** project administration, resources, supervision, writing – review and editing.

## Ethics Statement

This study was approved by the Ethics Committee of the Army Specialized Medical Center of the People's Liberation Army of China (Approval Number: 2022236). All research was conducted in accordance with the relevant guidelines and regulations.

## Conflicts of Interest

The authors declare no conflicts of interest.

## Transparency Statement

The corresponding authors Ya Chen and Ming Fu affirms that this manuscript is an honest, accurate, and transparent account of the study being reported; that no important aspects of the study have been omitted; and that any discrepancies from the study as planned (and, if relevant, registered) have been explained.

## Data Availability

The data that support the findings of this study are available from the corresponding author upon reasonable request.
